# Clinical Importance of Bone Matrix Damage Mechanisms for Fracture Prevention

**DOI:** 10.1007/s11914-021-00678-8

**Published:** 2021-04-20

**Authors:** Richard L. Abel, Richard Stavri, Marena Gray, Ulrich Hansen

**Affiliations:** 1 MSk Laboratory, Sir Michael Uren Hub, Department of Surgery and Cancer, Faculty of Medicine, Imperial College London, London, W12 0BZ UK; 2 Department of Mechanical Engineering, Faculty of Engineering, Imperial College London, London, SW7 2AZ UK

**Keywords:** Bone, Matrix, Biomechanics, Damage, Mineral, Fibril

## Abstract

**Purpose of Review:**

Bone matrix exhibits great complexity in its composition, structure and mechanics. Here, we provide a review of recent research articles and appraise the evidence that bone matrix quality is clinically important and possibly targetable for fracture prevention.

**Recent Findings:**

Deformation of mineralised collagen fibrils determines bone fracture mechanics. Slipping and separation at the mineral-fibril and fibril-fibril interfaces, respectively, are the structural mechanisms for plastic deformation and microcrack nucleation. Existing technologies for assessing bone tissue in vivo cannot measure matrix structure or fracture mechanics but have shown limited use in clinical settings for identifying fragility or following treatment outcomes based on composition.

**Summary:**

Matrix is biomechanically and clinically important, but the knowledge has not translated into clinical practice. The structural mechanisms by which a load is transferred from mineralised collagen fibrils to the whole bone via microcracking have been proven too complex to measure in vivo. The mineral-fibril or fibril-fibril interfaces might be suitable targets for diagnosing fragility or delivering molecules that reduce fracture risk by strengthening the mineral bonds while maintaining flexibility in the fibrils.

## Introduction

Understanding the composition, structure and mechanics of the matrix could be key to improving fracture risk prediction and prevention including identifying new treatment targets. Since the concept of bone quality appeared at the beginning of the 21st century [1, 2], a growing body of evidence has shown that strength and fragility are influenced by the interplay between mass, structure and material [3]. However, diagnostics or treatments based on bone material have not filtered through into clinical practice, even though mass and structure have been widely adopted in clinical decision making (e.g. DXA, CT etc.). It is important to take stock and review the evidence that bone matrix quality is clinically important and possibly targetable with respect to assessing and reducing fracture risk. This review will show that to translate our discoveries about bone matrix biomechanics, we will need to research tools and protocols for measuring the damage mechanisms which both contribute to, but also resist, fractures.

## Bone Matrix

**Fig. 1:**
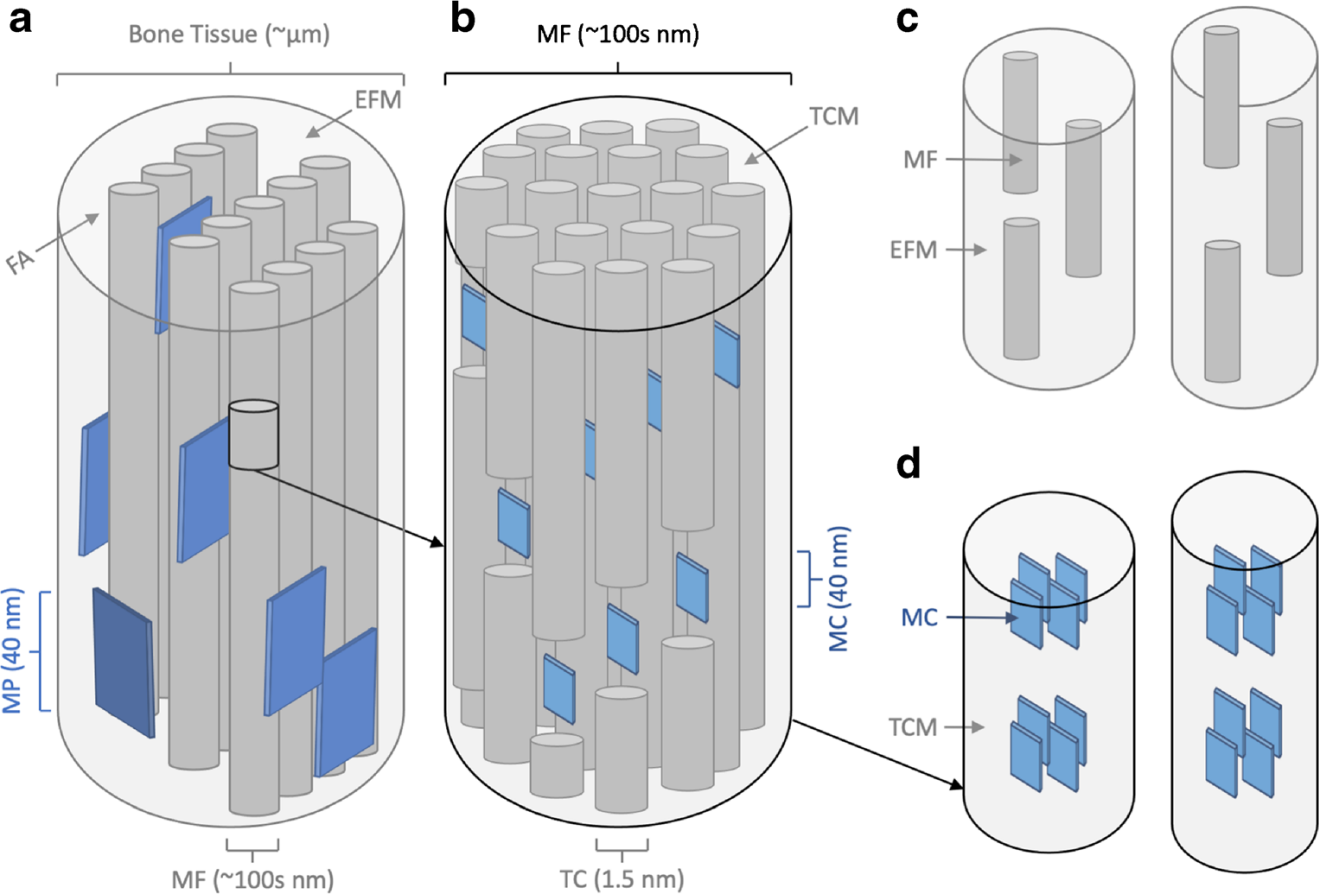
Bone nanostructure and deformation. **a** Bone tissue consists of mineralized collagen fibrils (MF) which are stacked in sheets of fibril arrays (FA). MFs are coated by extrafibrillar mineral platelets (MP) and surrounded by extrafibrillar matrix (EFM) which glues the MF together. **b** Each MF contains a matrix of tropocollagen molecules (TC) embedded with intrafibrillar mineral crystallites (MCs). **c** Tensile loads cause shear at the MF-MF interface the slipping and separation within the EFM. **d** Tension also causes shear between the MCs then slipping and separation within the tropocollagen matrix (TCM)

Essentially, bone tissue is a nanocomposite of mineralised fibrils (i.e. collagen fibrils with both extrafibrillar and intrafibrillar mineral apatite particles, but the ultrastructure of the mineralised fibrils is highly organised and complex (see Fig. 1a and b). Bone matrix is made from arrays of mineralised fibrils which are coated by extrafibrillar mineral platelets and glued together by an extrafibrillar matrix which fills the spaces in between (see Fig. 1a). Mineral platelets are formed from needle-shaped units, organised in stacks that span and integrate with adjacent mineralised fibrils [4]. With advancing tissue age, the mineralised fibrils mature and undergo post-translational modifications including the formation of the collagen crosslinks, which are covalent bonds between collagen fibrils. Each mineralised fibril is constructed from tropocollagen molecules, embedded with a semi-crystalline apatite mineral in the spaces between them (see Fig. 1b) [2].

Visualising the structure and quantifying the biomechanical behaviour of the matrix is technically difficult because the size of the fibril and mineral is on the order of nanometres (i.e. 10^−9^ m). To put nanoscale in perspective, begin by imagining an object the size of the sun and shrink it to the size of a football (i.e. a soccer ball!), and then shrink by the same factor again. Recently though, a few small studies applying atomic force microscopy and synchrotron diffraction imaging revealed insights into the biomechanical and clinical importance of the matrix.

## Biomechanical Importance of Matrix

The first was an investigation of nanoscale failure behaviour of cortical bone under stress using AFM which isolated the contribution of collagen and mineral structures to microcrack initiation [5••]. The authors applied an in situ tensile load to bovine cortical bone samples whilst simultaneously imaging the collagen-mineral matrix using AFM (atomic force microscopy). The images revealed the nanostructural mechanism in crack nucleation under stress (and likely also propagation). Initially, the elastic mineralised fibrils deformed before starting to separate within the extrafibrillar matrix; then, the mineral platelets deformed and started to separate. The separation at the fibril and fibril-mineral interfaces caused cracks to nucleate. The study is exceptionally useful because the images and data presented the first direct observations of the structural mechanisms linking nanomechanics of the matrix and micro-damage behaviour under loading: including the damage formation that can lead to a fracture.

The second was an investigation of the nanoscale failure of bone under stress using Synchrotron X-ray diffraction which isolated the contribution of mineral and fibril mechanics to tissue strength and fracture [5••]. The authors applied in situ tensile loads to samples of human trabecular bone from the proximal femur whilst simultaneously imaging deformation of the collagen and mineral matrix using Synchrotron X-ray diffraction at the Diamond Light Source (UK). The technique relies on the periodic structure of the mineral collagen matrix to measure strain under load. X-rays are diffracted by the spaces between mineral platelets, and the mineral crystal lattice. By radially integrating the patterns at two loads, it is possible to calculate strain using short-angle diffraction for the fibrils (i.e. SAXD) and wide-angle diffraction for the mineral (i.e. WAXD).

It is worth noting here that the synchrotron is a giant ring 738 m in circumference capable of accelerating electrons to near light speed, at which point the particles release energy in the form of monochromatic X-rays, useful for collecting low-noise images of human tissues. This low noise-to-signal is partly a consequence of the extremely high speed of the electrons (186,000 miles per second) wherein time slows and a fraction of a second in our perspective would be perceived to last days by the electrons, a phenomenon known in relativity as time dilation.

**Fig. 2:**
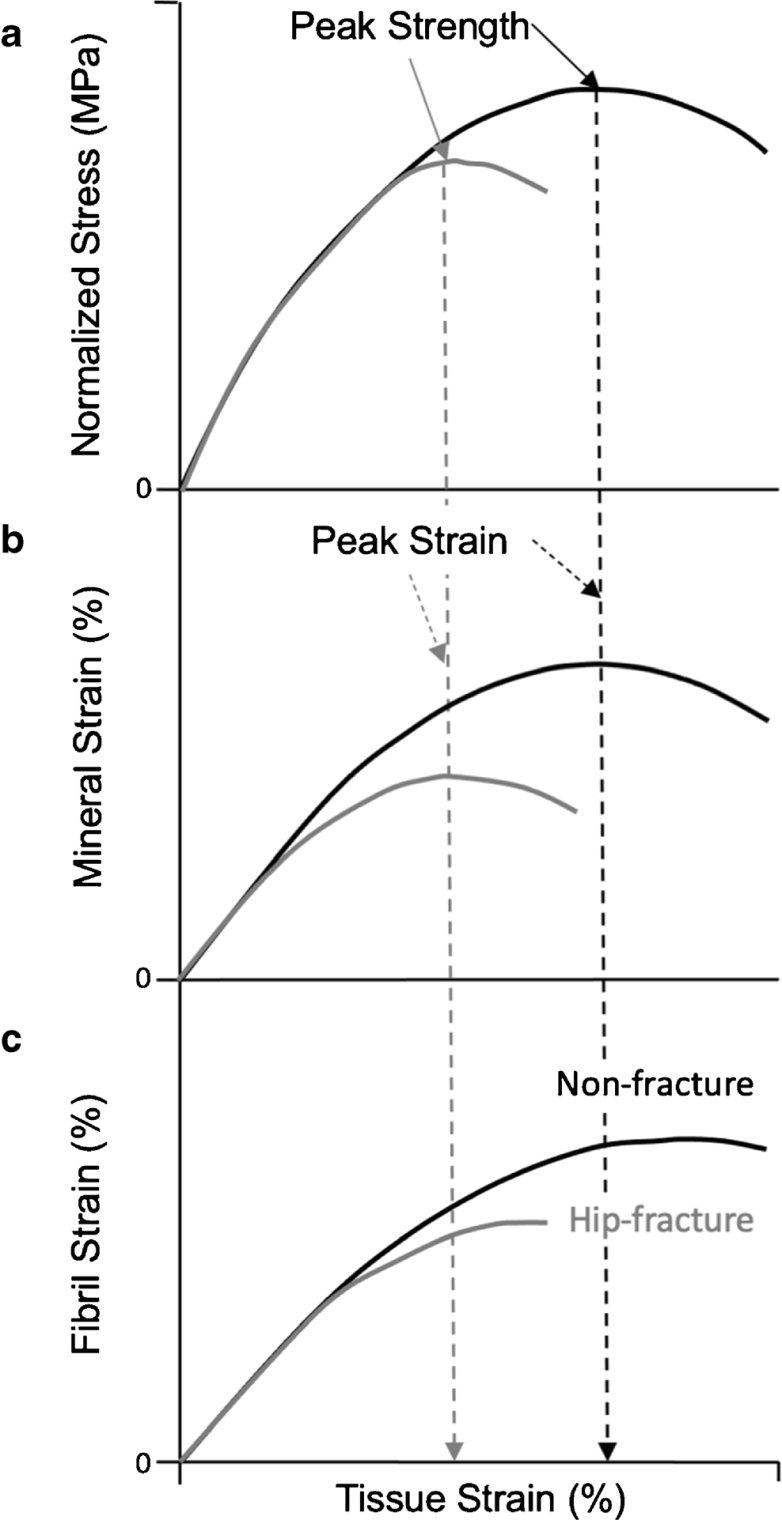
Differentiating the role of mineral and fibril strain in trabecular bone strength. **a** Peak tissue strength of trabecular bone cores coincides with the onset of **b** mineral sliding and decoupling from collagen fibrils (i.e., peak mineral strain). **c** But fibril strain plateaus after strength. Hip-fracture donors exhibit lower fibril, mineral, and tissue strain than non-fracture controls. Adapted from ^4^

After comprehending the dilation of space time, the authors used the diffraction images to compare the time evolution of tissue, mineral and fibril strains (Fig. 2). The peak tissue strength (i.e. ultimate stress) coincided with the peak mineral crystallite strain (i.e. at the same tissue strain), whilst peak mineralised fibril strain occurred afterwards (i.e. higher tissue strain) and plateaued until fracture. At peak strain, the mineral crystallite slips (see Fig. 1d) and then separates from the collagen matrix, so the data suggest that bone tissue fails when the mineral can no longer contribute to the load-carrying capacity of the bone. The peak crystallite mineral strain observed in normal ageing bone was about 0.15% which suggests that separation might start with a fracturing of the mineral. Peak mineralised fibril strain which corresponds to slipping and separation of mineralised fibrils (see Fig. 1c) plateaued in the region of plastic deformation (Fig. 2). The energy absorbed in making the bone tissue plastically deform was likely spent sliding and separating the fibrils. The authors suggested that the mineral fracture and separation dominated the peak material strength, and fibril separation absorbed energy during plastic deformation to stop the bone from fracturing. Hence, it is the mineral component ultimately that governs bone strength and failure, but fibrils were important in resisting fracture.

The ability of fibrils to resist fracture has been investigated in more detail using combined SAXD and SEM to image damage formation at the level of the mineralised fibril. The resulting images are as valuable for their artistic view of bone as much as the scientific analysis. Groetsch and colleagues [6] prepared mineralised collagen fibrils from turkey tendon using ion beam milling technique to create bone pillars (~ 6 μm *d* and ~ 12 μm *h*). The pillars were compressed with simultaneous SAXD; then, failure mode was interrogated by visualising damage using SEM. The SAXD data showed mineral particles strained before the fibrils, which supports the theory that mineral governs strain. The SEM images revealed that cracks were more likely to form in the extrafibrillar matrix or at the fibril-matrix interface. This damage within and between fibrils was characterised by incomplete separation with buckling of fibrils leading to ‘*bulging*’ and ‘*kinking*’ of the fibrils. The extent of buckling was proportional to plasticity (i.e. yield strain) but inversely proportional to strength (i.e. yield stress). Thus, the ability of collagen fibrils to resist crack formation (and propagation) could determine the ability of the tissue to absorb energy, but cracks could still form in the extrafibrillar matrix. To further complicate our understanding of failure modes, a study by Schwiedrzik and colleagues [7] reported that the location of crack formation was influenced by the fibril orientation. The authors used ion beam milling to prepare bone pillars (~ 5 μm *d* and ~ 10 μm *h*) from ovine bone and under compressive loading cracks formed at the interface between regions with different fibril orientation. Hence, the nanostructural mechanisms of damage deformation traverse the mineral, fibril, then extrafibrillar structure and the location of inhomogeneities could determine the stress field and therefore the location of the damage.

Fundamentally, these AFM, synchrotron and SEM studies agree that nanoscale slipping and separation are the key mechanism leading to microcracking and ultimately whole-bone fracture (Fig. 1). This is a distinct (structural) mechanistic pathway for a load as it transfers and scales up from the matrix to the level of the whole bone and could increase fracture risk by reducing fracture toughness [8]. The AFM study reports that collagen separation and slipping leads to mineral slipping and separation whilst the diffraction and SEM studies report the opposite, mineral disengagement and the subsequent onset of fibril sliding is one of the key mechanisms leading to fracture. The next step will be to combine AFM and diffraction imaging under in situ load to try and ascertain whether the fibrils strain and transfer load to the mineral or vice versa. Cortical and trabecular nanostructures may behave differently under load or collagen and mineral proportions might also affect nanoscale behaviour. Despite the discrepancy, the takeaway message from these studies is that the matrix plays a key role in resisting (or facilitating) crack nucleation and therefore in bone fragility and fracture.

### Clinical Importance

It, therefore, seems sensible to consider the clinical importance of the matrix in relation to bone strength and fragility. The synchrotron diffraction study [9••] went on to compare the human tissue, mineral, and fibril mechanics of trabecular bone samples from patients that had a fractured neck of femur (with either a bisphosphonate treatment history or without) versus fracture-naive controls (collected from a tissue donor bank). The max tissue strain, mineral strain and fibril strain were all significantly higher in the controls in comparison with the fracture donors. A similar human study conducted on cortical bone compared with tissue from osteoporotic (80.2 ± 9.4 years, *n* = 5) versus middle-age bone (34.8 ± 4.8 years, *n* = 5) [10]. Under tensile load, the fibril strain increased before plateauing (as a function of tissue stain). Osteoporotic bone exhibited a more pronounced plateauing of fibril strain and lower yield stress suggesting the tissue was less able to deform plastically. Combining the two studies, it is reasonable to conclude that low mineral and fibril strain (i.e. deformation) could provide a structural mechanistic origin for age-related and/or osteoporotic fragility fractures. Low nanoscale strain probably contributed to the hip fractures by reducing the material strength of the tissue, i.e. a reduction in the ability of bone tissue to bend and absorb energy lessened its resistance to cracking and fracturing during a trip or fall. The loss deformation causing singular or perhaps multiple cracks to nucleate, propagate and merge under continued applied stress until the fracture of a whole bone.

**Fig. 3:**
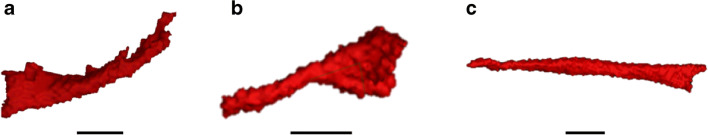
Synchrotron micro-CT reconstruction of micro-defects in bone tissue. **a** Microcrack, **b** partially resorbed crack, **c** osteoclastic trench. Scale bar 20 μm

Microcracks have often been implicated in fragility because of the potential to reduce bone strength. It is surprising then that in direct contrast to the work reviewed above, a recent study reported that hip fractures were not associated with an accumulation of damage [11••]. The authors applied synchrotron micro-CT to human trabecular bone samples and collected scans with a resolution of 1.3 μm, which was high enough to visualise both micro-cracks in the tissue and osteoclastic cavities formed by resorption. The density and size of micro-defects were compared between bone samples from patients that had a fractured neck of femur (with either a bisphosphonate treatment history or without) versus fracture-naive controls. Fracture donors tended to exhibit a lower micro-crack density than controls, but a significantly higher density of osteoclastic cavities. The existing cavities appeared to be ‘trenches’ in the bone that had probably formed when osteoclasts resorbed the microcracks as part of the remodelling process (Fig. 3). This finding highlights the complex role of the matrix in bone mechanics and fracture because of the interplay with metabolism.

The important discovery was that microcracks do not simply accumulate in bone, but potentially cause greater loss of strength by upregulating and focussing osteoclastic activity at specific sites. The osteoclastic resorption of cracks creating trenches which perforate bone tissue. There is some evidence that cavities then promote damage formation by causing stress risers that concentrate external loads and act as a nucleation point for new cracks, which in turn would promote additional resorption. As such material deficits in the bone matrix could create a vicious cycle of runaway resorption [12] that could lead to a great loss of structure and strength for a relatively small loss of mass, especially in the trabecular tissue when elements are perforated. To continue the central thread of this review, the effect of matrix quality (measured by peak mineral and fibril strain) on bone strength and fragility will depend on the complex interplay between microcrack formation, propagation and internal repair.

### Modelling Approaches for Testing Clinical Importance of the Matrix

Current technology does not allow the system of bone material, metabolism, damage and fracture to be interrogated experimentally because longitudinal studies of bone nano-mechanics and remodelling cannot be conducted simultaneously. In silico research might be useful for understanding the interactions between nano-scale behaviour and microcracks [13] and the relationship between cracks and cavities [14]. A recent study by Alizadeh and colleagues used experimental data to build a multiscale finite element analysis (FEA) of cow bone which successfully predicted the stress at which mineralised collagen fibrils separated [13]. The Alizadeh model also predicted that shearing between nanoscale collagen and fibril reduces the max load or energy to break bone tissue samples (i.e. the yield stress of tissue). In a similar approach, Easley and colleagues built a finite element model to test the biomechanical effect of trench-like resorption cavities by virtually perforated trabecular structure [14]. Under simulated load, any trenches added to high strain regions caused a greater reduction in strength than those added to low strain regions, and the loss of strength was accentuated by low bone volume. There is potential then to apply finite element analyses to study the scale transition of load transference from the mineral/fibril to the tissue level, whilst accounting for the formation of microdamage and the effects of resorption. Such a sophisticated computer model could be used to test the effect of ageing or disease from the matrix up to the tissue level and identify targets for novel diagnostics of therapies.

### Targeting Bone Matrix

Given the apparent importance of matrix in initiating and propagating fractures, it seems very plausible that mineral-fibril mechanics could be targeted to assess bone fracture risk or monitor treatments outcomes. Before new diagnostics could be developed or tested, it will be crucial to find protocols for measuring matrix mechanics in vivo (or proxies such as structure and composition). There are two techniques which have gained popularity including reference point indentation (RPI) [15] and Raman spectroscopy [16]. Until now with a few notable exceptions, the instruments have been tested ex vivo with conflicting results. Clinical studies have reported that the instruments can distinguish bone samples from fracture donors versus controls and track the matrix changing in response to common treatments. However, biomechanical studies do not report a strong correlation between indentation or Raman data with bone mechanics (i.e. strength and stiffness). Until this paradox is resolved, it will be difficult to persuade clinicians and patients to adopt these technologies into clinical practice (and rightly so).

RPI is an engineering technique for pressing a hard-tipped material into another material with a known force to measure hardness. Bone studies typically use a microscopic needle to indent a bone surface using either cyclic or impact loading. Benchtop (e.g. BioDent) and in vivo systems (e.g. OsteoProbe) are available to researchers. BioDent applies cyclic reference point indentation (with a reference probe). OsteoProbe purports to measure bone quality via impact micro indentation (high loading rate) using the ‘bone mechanical strength index’ or ‘BMSi’ by creating 2 indents at 10 N and 30 N load and calculating the depth between.

OsteoProbe was recently used to assess the effect of ageing [17] and treatment [18] on the cortical bone matrix. In a study of cadaveric human tibia, 20 females ranging from 53 to 97 years were assessed and correlations between BMSi measures and cortical geometry ranged from very weak to very strong (*r*^2^ = 0.03 to 0.98) [17]. BMSi was also independent of age with similar values in senior (53–69 years) and older (70–97 years) age groups. In a separate study, OsteoProbe was used to follow up and compare a group of patients prescribed antiresorptive treatments (i.e. bisphosphonates or denosumab) versus a control group prescribed supplements (i.e. vitamin D) [18]. In the antiresorptive group, BMSi increased significantly from baseline to 2 years whilst the supplement group did not change. Potentially then, in vivo indenters could be used to track some measure of bone quality, but it is not clear what indentation captures.

Many proponents of the RPI technique point out that indentation can create microcracks and the depth could measure fracture toughness. This has not been borne out by biomechanical studies using benchtop systems including a BioDent 1000 system (manufactured by the same company as OsteoProbe). The cortical bone surface of rat and dog femora were indented (5 N for 10 cycles at 2 Hz) and the BioDent measured the first indentation depth and last indentation depth to calculate the increase in depth over the loading cycle in rat femora and dog ribs [19]. The increase in depth was moderately and negatively correlated with apparent material toughness measures obtained using 3-point bending (*r*^2^ values ranging from 0.50 to 0.57). Similar studies based on human bone from the proximal femur reported that indentation measures with the BioDent were only weakly positively correlated *(r*^2^ = 0.33) with compression modulus (i.e. stiffness) collected using standard testing procedures in an Instron loading rig [20]. The authors noted that the weak correlation could reflect the heterogeneity of tissue because indentations in various regions of the bone surface may have sampled regions with varied osteonal structure or porosity.

The RPI was an interesting approach for studying the mechanics of the matrix directly. An alternative and less direct approach to capturing the matrix has been to assess the composition, on the basis that composition influences structure and mechanics. Raman spectroscopy uses a laser to pass energy into molecular bonds causing them to vibrate and then measures the shift in wavelength of the energy profile to assess the composition of bone. Bone studies typically use benchtop systems to assess bone tissue samples but one in vivo system (i.e. SORS) has been developed [21]. The main drawback of the Raman technique is that the spectra outputs are difficult to interpret especially in relation to mechanical properties. The spectra profile both the mineral and collagen components of bone tissue including the phosphate, carbonate and collagen (i.e. amides). Data is usually extracted from the spectra via the calculated ratio of the peak heights. Most Raman publications are aimed at a specialist audience and the articles which are aimed at bone scientists tend to underplay the limitations of the technique.

A notable and recent paper which can be recommended investigated cadaveric human femora and tested whether the Raman spectral data correlated with fracture, measured using 3-point bending and micro-CT imaging of crack growth [22]. The correlations between spectral ratios were low to moderate (*r*^2^ values ranging from 0.25 to 0.47). The results suggest that benchtop Raman microscopes have limited potential for assessing fracture toughness of bone. Therefore, the in vivo systems which must also pass a laser through soft tissues will have an even lower capability of measuring the bone composition and fracture toughness. However, like the RPI technique, several recent publications have reported some potential for using Raman to identify fractures, the effect of comorbidities and treatment outcomes.

A Raman analysis and comparison of trans iliac biopsies from fractured versus non-fractured female donors (matched for BMD) reported that spectra did not distinguish the groups but fracture donors exhibited significantly lower enzymatic collagen cross-link content (i.e. pyridinoline) [23•]. A similar but separate study comparing transiliac biopsies from osteoporotic donors supplemented with calcium and vitamin D for 3 years versus treatment-naïve also reported that spectra did not distinguish the groups. However, supplementation was associated with lower mineral/matrix and higher enzymatic collagen cross-link content (pyridinoline) at actively forming trabecular surfaces [24•].

### Treatment Targets

Promisingly, the studies reported that fractures were associated with higher cross-link content (i.e. both enzymatic and AGE) [23•] whilst the treatment was associated with lower content (i.e. enzymatic) [24•]. The finding takes us back to where this review started. The cross-links could be biomechanically important by limiting strain, slipping or separation at fibril-fibril or mineral-fibril interfaces. Very speculatively, it might be possible to propose that molecules which increase the flexibility of the collagen-mineral matrix could reduce fragility and the risk of fracture during a traumatic load. Flexibility could be modulated by reducing the number of collagen cross-links at the fibril-fibril interfaces or strengthening the bonds at the mineral-fibril interfaces. Delaying the onset of slipping and separation (especially at the mineral-fibril interface), thereby increasing the capacity of the tissue to absorb energy (both before reaching the maximum load-carrying capacity and during plastic deformation), might similarly be deemed an important target.

Flexibility in the mineral component could also be modulated by altering the structure, size or composition (including imperfections). Indeed, the frontline treatments for reducing fracture risk (i.e. bisphosphonates) might already cause such effects. Although fracture reduction efficacy is usually attributed to an increase in mineral density, there will be effects on nanostructural mechanics and damage mechanics. The effect is not clear though because synchrotron diffraction studies of mineral strain (which were described above) have reported that treatment is associated with a reduction in strain in trabecular tissue [9••] but an increase in strain in cortical tissue [10]. These gaps in our knowledge need to be filled to optimise current therapies and develop new interventions.

### Matrix Organisation

While mineral and collagen have been investigated in detail, the contribution of other components to nanoscale damage mechanisms remains unclear. For example, there is evidence that the major non-collagenous proteins including osteocalcin (OC) and osteopontin (OPN), as well as water and mineral structure, affect cracking and fracture at higher scales. Poundarik and colleagues [25] applied laser microdissection and ELISA to strained human bone samples and reported that microcracks (~ 100 s nm) initiate around the OC and OPN complexes which bind the mineral and collagen together. The author suggested that the initial damage around OC and OPN could lead to collagen fibril shear, sliding and separation. Further, fracture tests on bones from OC or OPN knockout mice revealed lower toughness and less diffuse damage (i.e. lower plasticity) in comparison with controls, demonstrating the effects of OC and OPN at higher length scales. Importantly, the loss of toughness associated with OC and OPN deficiency is a material deficit which is independent of bone mass [26].

Both OC and OPN might affect nanoscale material properties and damage mechanics via bioregulation of the bone mineral and subsequent effects on the interaction with collagen. Strong evidence in support of this theory comes from another related knockout mice study by Poundarik and colleagues investigating the effect of OC and OPN on mineral morphology and composition [27]. The mineral thickness and orientation were captured using benchtop X-ray diffraction, morphology with SEM and trace element composition with wavelength dispersive spectroscopy (i.e. magnesium, calcium, sodium and sulphate ratio). Diffraction spectra revealed that loss of OC and OPN was associated with reduced crystal thickness and alignment and altered trace element composition. OC had a stronger association with crystal morphology and OPN with composition. Loss of OC has also been reported to reduce other trace elements including carbonate [28]. Any such changes in morphology and composition including trace elements are likely to affect the ability of mineral the bear load before sliding and separating from the fibrils. The specific effects have not yet been established, but experiments based on synthesized mineral crystals reported that smaller or more carbonated mineral crystals exhibit increased microstrain in comparison crystals that were larger or possessed a higher phosphate content [29].

Fibril mechanics are also likely affected by the organisation of the matrix, and a key effect might come from bound water. Water residing within gaps the bone matrix (< 4 Å) might increase toughness [30] by improving fibril strain [31]. A recent experimental study by Samuel and colleagues [32] revealed remarkable complexity. Bone samples from human femora were analysed using the now-familiar combination of Synchrotron X-ray diffraction and in situ progressive loading to capture mineral and fibril deformations in both hydrated and dry bone. The key finding was that the collagen and mineral strain was higher in hydrated tissue when compared with dry (as a function of tissue strain). Further, in the wet state, the mineral particles experienced higher stress than the dry, suggesting that dehydration impaired strain transference to/from the collagen, which could promote sliding, separation and failure at the interface. Intriguingly, wet tissue exhibited a loss of mineral strain post-yield, whilst dry tissue did not. In contrast, fibrils did not exhibit such strain relaxation post-yield irrespective of hydration status or loading mode. The authors reported that water content might therefore affect ultra-structural mechanics by altering the matrix nanostructures surrounding the mineral. A great example of data-led discovery science which shows that we have a long way to before we understand the role of fibril or mineral in nanoscale damage mechanisms including the key step of crack and fracture imitation in bone matrix. This research investigating the complex relationship between matrix organisation, nanomechanics, damage and material properties is vitally important and can already be developed and expanded with existing technology and techniques.

## Conclusion

The research reviewed in this paper is strong enough evidence to conclude that bone matrix is both biomechanically and clinically important and, therefore will (at least in principle) be targetable for assessing and reducing fracture risk. Translation of this knowledge has so far eluded scientists because of the complexity of the matrix composition, structure and mechanics. The key to understanding how the matrix affects whole bone strength and fragility is likely to be the damage mechanisms that allow mineral/fibril slipping and separation and enable the nucleation then of propagation of cracks that cause a whole bone fracture. Existing technologies purporting to measure mechanical properties or composition might collect some clinically relevant data but have not translated into clinical settings, in part due to the lack of mechanistic data. We propose that the most likely target for a diagnostic predictor of fracture risk would be the strength of the bond at the collagen-mineral interface, which determines the maximum material strength of the bone, whilst collagen crosslink content could be a suitable target for following existing antiresorptive treatment outcomes. Both the fibril-fibril and mineral-fibril interfaces (and components of the matrix which affect the interactions) might be suitable targets for molecules that reduce fragility and fracture risk by increasing the load-carrying capacity of the tissue and the ability to absorb energy via plastic deformation, preventing fractures nucleating and further propagating in bone tissue.
